# Scarlet Fever

**Published:** 1889-10

**Authors:** 


### SCARLET FEVER.

Dr C. S. Harris, of Rome, Ga. writes: According to request I send you my treatment for scarlet fever. I have used it for thirty years. Used it in three epidemics in Tennessee.

Take six grs. sulph. zinc, divide into six powders, 1 gr. each. Give one every three hours. If your case is better after taking the six powders, then give one every six hours.

About five years ago we had scarlet fever here. Dr. Hoyt requested me to let him know when I had a fullly developed case, that he wanted to see it with me.

I did so. It was a child about ten years old. We found the child with a burning fever; temperature 105; throat very much inflamed, scarlet rash; pulse full, strong and rapid; tongue coated.

I gave her 10 grs. sulph. zinc every three hours until she had taken thirty grs. It produced slight nausea, then gave her the 1 gr. doses. Dr. Hoyt saw the case with me the next day, found the child doing well. Scarlet rash had disappeared; skin moist; tongue clean; pulse natural, child wanted to eat. Next day discharged her. Two more cases in the same family yielded readily to the same treatment.

I gave the 10 grs. to see how far I could go without producing emesis, knowing that I was there to watch it. I would not advise that much, prefering the six gr. doses, which is safer, and sufficient to accomplish the cure.

